# Experimental and Computational Evaluation of Chloranilic Acid as an Universal Chromogenic Reagent for the Development of a Novel 96-Microwell Spectrophotometric Assay for Tyrosine Kinase Inhibitors

**DOI:** 10.3390/molecules26030744

**Published:** 2021-01-31

**Authors:** Ibrahim A. Darwish, Hany W. Darwish, Nasr Y. Khalil, Ahmed Y. A. Sayed

**Affiliations:** 1 Department of Pharmaceutical Chemistry, College of Pharmacy, King Saud University, P.O. Box 2457, Riyadh 11451, Saudi Arabia; hdarwish@ksu.edu.sa (H.W.D.); nkhalil@ksu.edu.sa (N.Y.K.); ahmedyahia@ksu.edu.sa (A.Y.A.S.); 2 Department of Analytical Chemistry, Faculty of Pharmacy, Cairo University, Kasr El-Aini St., Cairo 11562, Egypt

**Keywords:** tyrosine kinase inhibitors, chloranilic acid, charge-transfer reaction, 96-microwell spectrophotometric assay, high-throughput pharmaceutical analysis

## Abstract

The tyrosine kinase inhibitors (TKIs) are chemotherapeutic drugs used for the targeted therapy of various types of cancer. This work discusses the experimental and computational evaluation of chloranilic acid (CLA) as a universal chromogenic reagent for developing a novel 96-microwell spectrophotometric assay (MW-SPA) for TKIs. The reaction resulted in an instantaneous formation of intensely purple colored products with TKIs. Spectrophotometric results confirmed that the reactions proceeded via the formation of charge-transfer complexes (CTCs). The physical parameters were determined for the CTCs of all TKIs. Computational calculations and molecular modelling for the CTCs were conducted, and the site(s) of interaction on each TKI molecule were determined. Under the optimized conditions, Beer’s law correlating the absorbances of the CTCs with the concentrations of TKIs were obeyed in the range of 10–500 µg/well with good correlation coefficients (0.9993–0.9998). The proposed MW-SPA fully validated and successfully applied for the determination of all TKIs in their bulk forms and pharmaceutical formulations (tablets). The proposed MW-SPA is the first assay that can analyze all the TKIs on a single assay system without modifications in the detection wavelength. The advantages of the proposed MW-SPA are simple, economic and, more importantly, have high throughput.

## 1. Introduction

Cancer is the world’s second major cause of death among males and females. This reflects ~9.6 million deaths in 2018 (~13% of all deaths). Cancer deaths worldwide are expected to increase significantly, with ~13.1 million deaths in 2030 [1]. According to many reported studies, cancer is a growing problem [2,3,4] and therefore it continues to spread worldwide, with a tremendous burden on individuals, families, communities and healthcare systems, both mentally and socially [5]. The majority of health care systems in developed countries are little prepared to deal with this challenge, and many cancer patients globally will not have the privilege of early treatment. In these countries, nearly two-thirds of cancer patients die [6]. There is evidence that shows that in countries where national health services are well-developed, strategies such as early detection, treatment and survivorship care are improving the survival rates of many types of cancers [1,6].

There are a number of treatment options for cancer such as surgery, radiation therapy and/or chemotherapy. For treating localized cancers, radiation therapy and surgery are preferred, while chemotherapy is considered safe for systemic cancers. Although chemotherapeutic agents exert their cytotoxic effect by way of interfering with the synthesis or the function of proteins and other needed cellular biomolecules, they also attack RNA, DNA and other vital proteins. Consequently, one must be careful when administering chemotherapy, as it has major side/toxic effects since it is capable of killing cells. A perfect recorded chemotherapy would be highly selective and specific to sick or cancerous tissues, while leaving healthy ones untouched. Unsurprisingly, the reality always differs from expectations, as most chemotherapies are heavily toxic, particularly for short-lived cells [7].

Recent developments in life science research in recent years have led to increased understanding of signaling transductions in tumor cells, apoptosis triggering, cellular interactions and other critical processes [8]. In addition, these chemotherapeutic drugs may be highly selective for DNA and other cellular targets that present in cancer cells as well as normal cells. The use of major intracellular signal transduction enzymes relevant to the differentiation and spread of cancer cells as drug testing objectives has become a hot area for medical research and development of antitumor drugs, and the continued improvement of effective, safe and specific new targeted anticancer drugs [9].

Tyrosine kinase (TK) is an enzyme that is responsible for transferring phosphate group from adenosine triphosphate (ATP) to the tyrosine residues of certain proteins inside the living cell [10]. An abnormality in TK could lead to a series of body diseases. Earlier investigations have demonstrated that there are TK activities for more than half of mutated proto-oncogenes and oncogenes, and the irregular expression of these genes can contribute to abnormal cell proliferation and eventually cancer [11]. Additionally, in conjunction with tumorigenesis, neovascularization and chemotherapy resistance, an abnormal expression of TK has been observed [12]. For that reason, targeting TK has attracted even more interest for the pharmaceutical industry for developing new chemotherapeutic drugs. Researchers and pharmaceutical organizations have been given high priority to developing tyrosine kinase inhibitors (TKIs) that may affect unique molecular pathways [13]. In 2001, the Food and Drug Administration (FDA) quickly approved the first targeted TKI drug imatinib and stimulated new energetic thought to cancer treatment [14,15,16]. To date, the FDA has approved over 20 TKI drugs [17]. These drugs are characterized by high effectiveness and low drawbacks and dominate the management of various types of cancer [10,11,12,13,14,15,16,17] compared to traditional cytotoxic antinineoplastics, some of which have become the foremost cancer treatment. The safety and efficacy of TKI medications is principally based on their corresponding pharmaceutical formulation quality, including drug content and uniformity.

For the quality control (QC) of TKIs, it is absolutely necessary to use proper analytical techniques. The analytical methods mentioned in the literature for QC of TKIs in their dosage forms are HPLC and HPTLC [18,19,20,21,22,23,24,25,26,27], voltammetry [28], spectrofluorometry [29,30,31,32] and spectrophotometry [33,34,35,36,37,38,39,40,41,42,43,44]. The most accessible methodology is spectrophotometry, which is quite easy, the least expensive and available in most quality control laboratories. Nevertheless, almost all of these spectrophotometric assays for TKIs are based on measurements of native UV absorption, which are not selective [33,34,35,36,37,38,39]. Few visible-spectrophotometric methodologies with varying chromogenic reagents have been developed for assaying TKIs [40,41,42,43,44]. Even worse, these methods incorporate laborious extraction steps and utilize large volumes of toxic organic solvents [45,46,47,48]. Furthermore, due to the differences in chemical structures of analyzed TKIs, these assays were individually developed. Besides, these assays have limitations regarding throughput, as they use traditional spectrophotometry.

**Figure 1:**
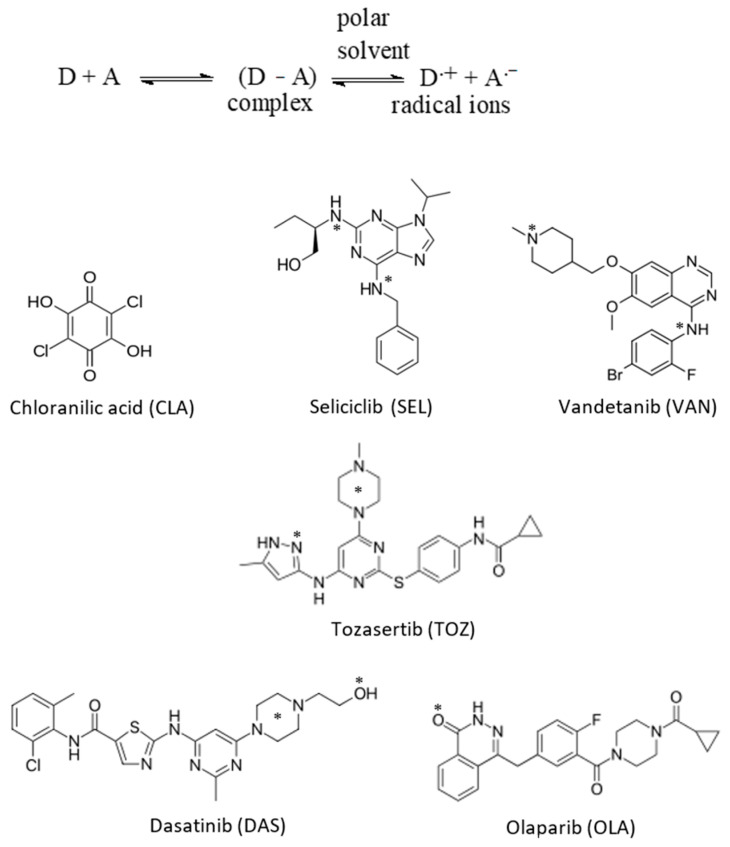
The chemical structures of chloranilic acid (CLA) and the investigated tyrosine kinase inhibitors (TKIs) with their abbreviations. Asterisks symbol (*) denotes the electron-donating sites of interactions of TKIs with CLA and the charge-transfer complexes (CTCs) formed.

From the presented information, establishing a universal spectrophotometric method for assaying any TKI irrespective of its chemical structure would be of great benefit and convenience. This research is directed to establish a novel 96-microwell-based spectrophotometric assay (MW-SPA) that could be applied in QC laboratories for reliable and accurate determination of any TKI. The proposed approach is based on formation of charge-transfer complexes (CTCs) between TKIs and chloranilic acid (CLA). The proposed procedure was established and validated for five TKIs. These TKIs were seliciclib (SEL), vandetanib (VAN), tozasertib (TOZ), dasatinib (DAS) and olaparib (OLA); their chemical structures are given in Figure 1. Their chemical names, molecular formulae and molecular weights are given in Table 1.

## 2. Results and Discussion

### 2.1. Strategy and Design of the Study

Our selection for TKIs as target analytes is due to their therapeutic relevance and the need to develop a globalized analytical methodology for their quantification in their dosage forms regardless of their differences in chemical structure. By virtue of its simplicity and spreadability in almost all analytical laboratories, spectrophotometry was adopted for assaying TKIs in the current work. The CT reaction of the investigated TKIs were examined in this section on the basis of their expected high electron-donating capability due to the presence of multiple potentially electron-donating sites on the chemical structures of all TKIs (Figure 1); these sites may form CTCs with electron-acceptors. Previous research, which include CT reactions with several polyhalo-/polycyanoquinone electron acceptors, have shown that CLA is by far the most reactive acceptor because its reactions are instant at room temperature [49,50]. This is the reason why CLA was selected from other acceptors for the current work. Since traditional CT-based spectrophotometric methods have a restricted throughput and utilize large volumes of organic solvents that are costly and, more crucially, cause toxicity to analysts [45,46,47,48], this study was dedicated to developing a spectrophotometric assay for TKIs that is free from these demerits. Accomplishing this goal was achieved by performing a CT reaction between TKIs and CLA in 96-microwell assay plates and recording the color intensity by a microplate absorbance reader. This technique employs low volumes of organic solvents and offers a high-performance analysis that serves the needs of QC laboratories, as it allows analysts to rapidly perform huge numbers of samples and to obtain large datasets that would otherwise exhaust assets in terms of costs, effort and time [50,51].

### 2.2. UV–Visible Absorption Spectra and Band Gap Energy

**Figure 2:**
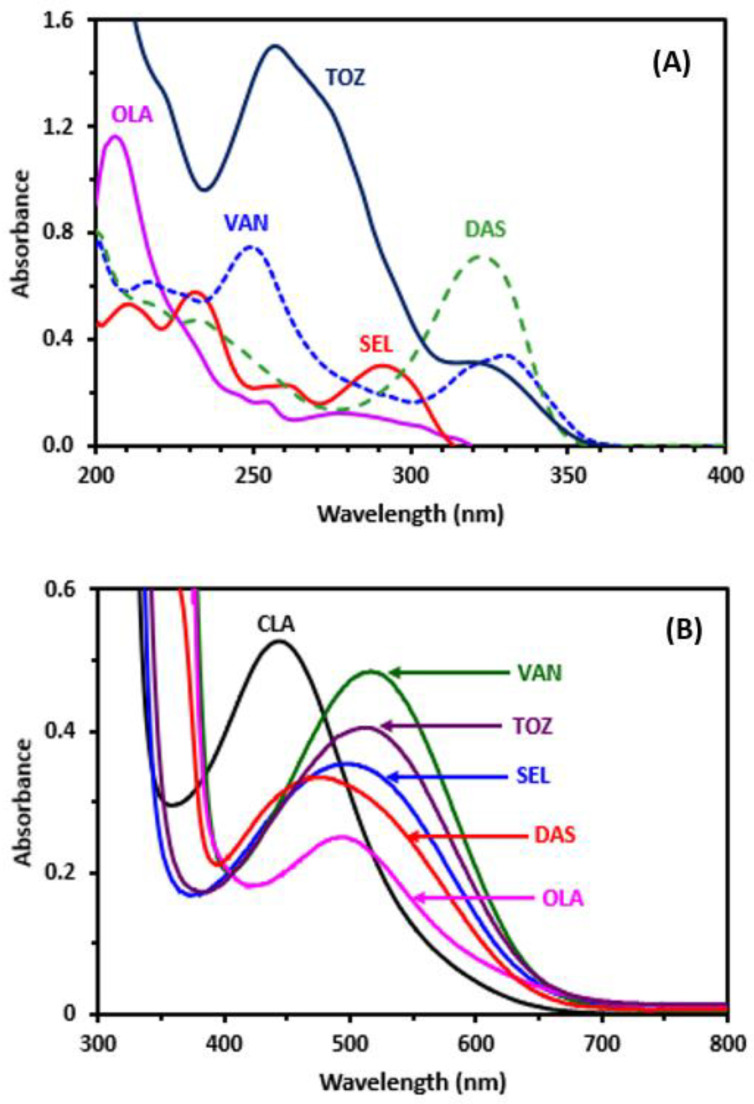
Panel (A): Absorption spectra of TKIs (10 µg/mL). Panel (B): absorption spectra of CLA (0.1%, *w/v*), and its reaction mixtures with TKIs (100 µg/mL). All solutions were in methanol.

UV–visible absorption spectra of various solutions of TKIs were measured in the range of 200–400 nm. The spectra exhibited various shapes, maxima and molar absorptivities. This is due to variability in their chemical structures (Figure 1). Nevertheless, none of the studied TKIs exhibited any reading above 360 nm (Figure 2). When TKI solutions were mixed individually with a yellow–orange color solution of CLA (its (λ_max_ of 444 nm), the solutions changed to purple color and the corresponding absorption spectra shifted toward larger wavelengths of both CLA and TKIs (Figure 2). Table 2 depicts all (λ_max_) and the molar absorptivities (ε) for all TKIs. It was confirmed that the new absorption bands of TKI–CLA product were generated as a result of the reaction between CLA and TKIs, and the absorption intensities depended on the concentrations of TKIs. The TKI–CLA absorption spectra produced was shown to have the same form and pattern as the CLA radical anion formed as a result of the reduction procedure, as published previously [49,52]. The reaction was therefore suggested to be a CT interaction between TKI (electron donor (D)) and CLA (electron acceptor (A)) and the reaction initiated in methanol (polar solvent) to form the CTC (D–A). This complex was then disassociated by the ionizing power of methanol and formed the CLA radical anion, as shown below.

**Figure 3:**
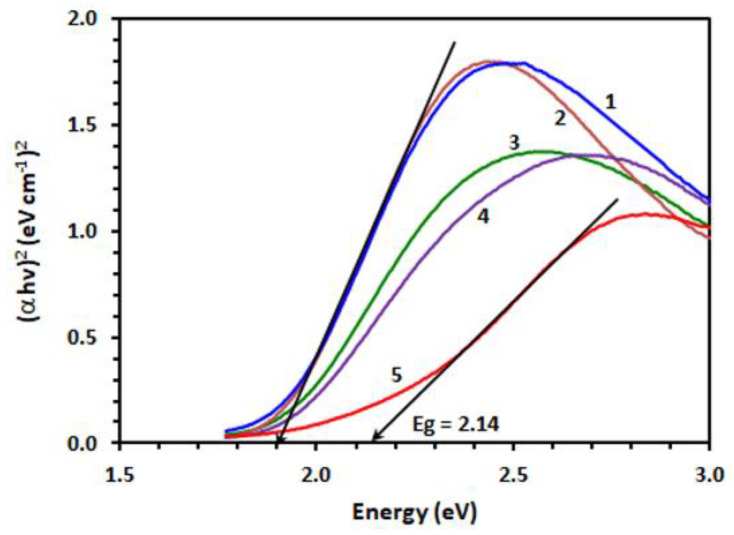
Tauc plots of energy (h) against (αh)2 for CT complex of CLA with TOZ (1), VAN (2), SEL (3), DAS (4) and OLA (5).

It is well known that CLA has three different ionic forms depending on the pH value. The first form that occurs at low pH is the neutral yellow–orange form (H_2_A), while the second form is the purple form (HA^−^), which is stable at pH = 3, and lastly the third form is the pale violet form (A^2–^), which is stable at high pH [53]. It was observed that the resulting products of all the investigated TKIs with CLA were purple; accordingly, the form HA^−^ was the concluded form of CLA that is involved in the current reaction. Additionally, there was other evidence for the suggested reaction, namely the disappearance of the formed purple color upon addition of mineral acids to the reaction mixtures. All of these findings support the development of the CTC between CAL and TKIs. The band gap energy (Eg) is the smallest required energy for excitation of an electron from the lower energy valence band into the higher energy band to participate in formation of a conduction band [54]. In order to compute Eg values, a Tauc graph was created from the absorption spectra of the TKI–CLA complexes by drawing energy values (hυ, in eV) against (αhυ)^2^ (Figure 3). Eg values were attained by extrapolation of the linear segments of the plots to (αhν)^2^ = 0 [55]. The results showed that Eg values ranged from 1.90 to 1.92 eV for all the investigated TKIs (Table 2). These results illustrate the easiness of electron transfer from TKI to CLA and the formation of CTC absorption bands.

### 2.3. Optimizing Conditions for CT Reaction of CLA with TKIs

In order to pick the best solvent for color development optimum reaction conditions, various solvents with different dielectric constants [56] and polarity indexes [57] were tried and the absorption spectra reported. These solvents were acetonitrile, methanol, ethanol, acetone, propanol, butanol, dichloroethane, dichloromethane, chloroform, diethyl ether, benzene and dioxan. Small shifts were noticed in the λ_max_ values, as well as changes in molar absorptivity (ε) values. As anticipated, the interactions in more polar solvents that possess large dielectric constant values, such as methanol and acetonitrile, provided ε values when compared with less polar solvents, such as diethyl ether and chloroform. This was attributed to the complete transfer of electrons from the TKI molecule to CLA in polar solvents; hence, methanol was chosen throughout this work.

### 2.4. Association Constants and Free Energy Change for the CTC of TKI–CLA

**Figure 4:**
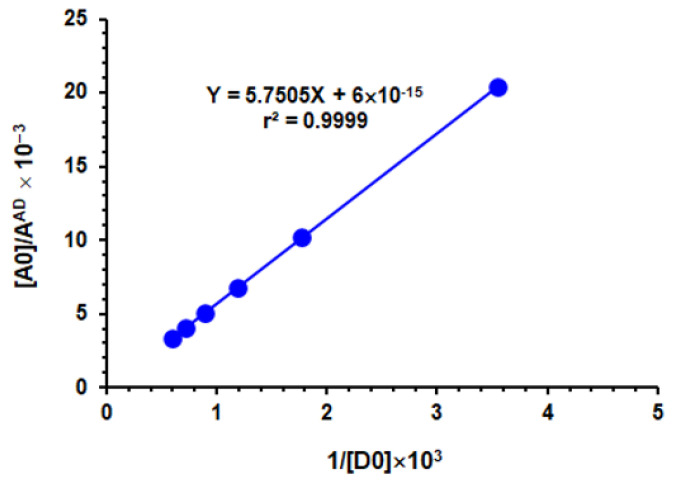
Benesi–Hildebrand plot of the CT complex of CLA with SEL, and the linear fitting equation with correlation coefficient (r). [A_0_], A^AD^ and [D_0_] are the molar concentration of CLA, absorbance of the complex reaction mixture and molar concentration of SEL, respectively.

The Benesi–Hildebrand method [58] was applied for calculation of the association constants (K_c_) at room temperature (25 ± 2 °C) and at the λ_max_ of the formed TKI–CLA complexes. As shown in Figure 4 as a representative example, straight lines were obtained from which the association constants of the CTC were computed. The obtained values of the association constants ranged from 1.13 × 10^8^ to 2.3 × 10^8^ L mol^−1^, as depicted in Table 2.

The standard free energy change (ΔG^0^) of the CTC were calculated using the following formula:

ΔG^0^ = −2.303 RT log K_c_

where ΔG^0^ is the standard free energy change of the complex (KJ mol^−1^), R is the gas constant (8.314 KJ mol^−1^), T is the absolute temperature in Kelvin (°C + 273) and K_c_ is the association constant of the complex (L mol^−1^). ΔG^0^ values were found to be comparable to all TKIs (~ 4.72 × 10^4^ J mol^−1^). These values proposed the easiness of the TKI interaction with CLA, as well as the stability of the formed CTCs [59].

### 2.5. Molar Ratio of the Reaction, Molecular Modelling of CTCs and Determination of the Sites of Interaction

**Figure 5:**
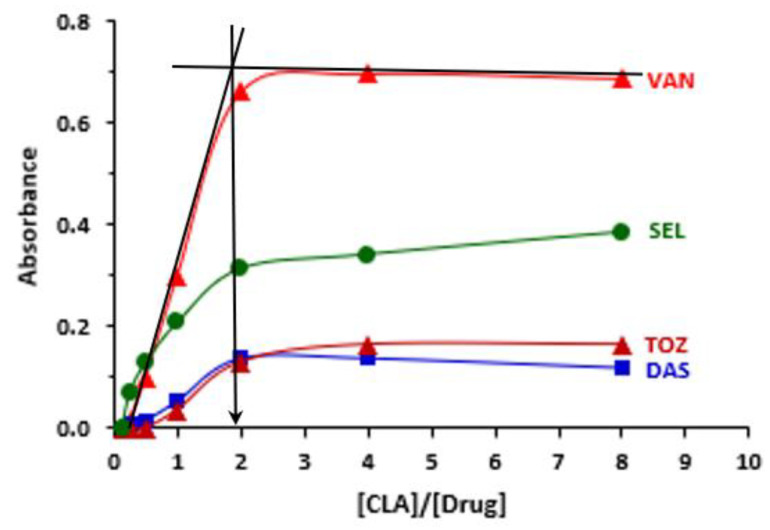
Plot of absorbance versus molar ratio of [CLA]/[Drug] obtained from reaction mixtures containing a fixed concentration of drug and varying concentrations of CLA. The mole ratio corresponds to the point of intersection of the tangents of straight-line portions of the plots (as shown for VAN). Measurements were carried out at 490 nm.

The published spectrophotometric titration methodology [52] was adopted to find out the molar ratio of TKI to CLA, and it was found to be 1:1 for OLA and 1:2 for SEL, VAN, TOZ and DAS (Figure 5). The molecular modelling and energy minimization of the TKI molecules and the CTC were performed, and the electron density of each atom was computed for allocation of these sites from the multiple electron-donating sites that exist on TKI molecules (Figure 1).

**Figure 6:**
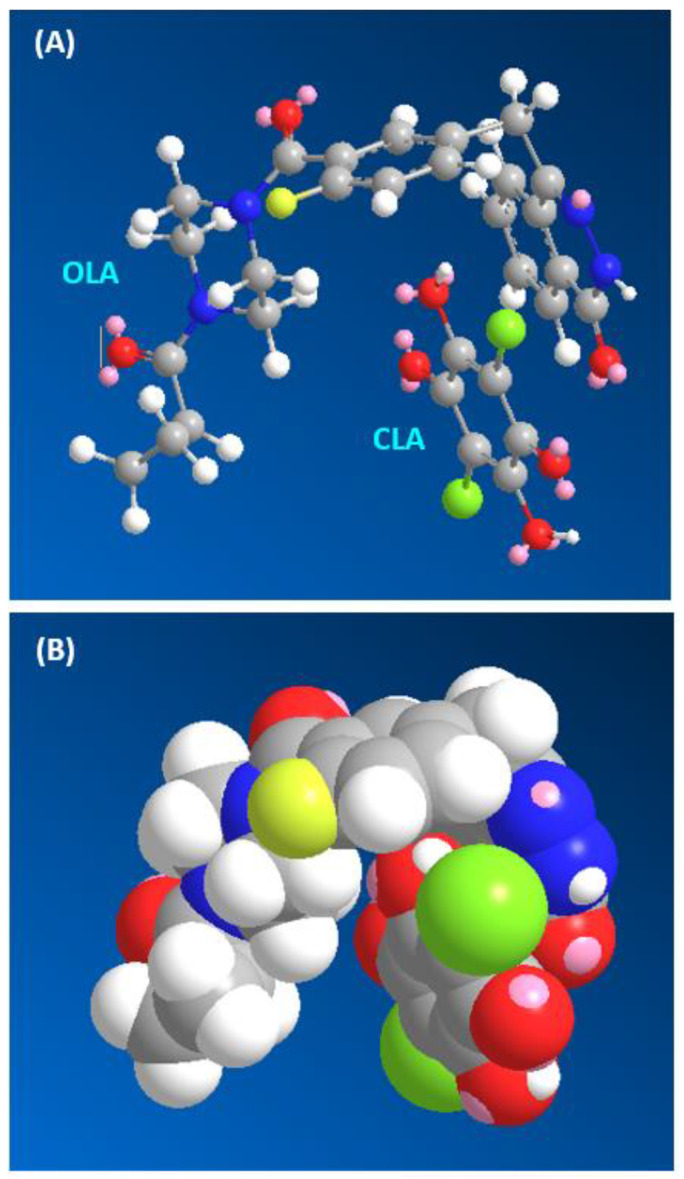
Energy-minimized CTC of CLA with OLA in the conformational (**A**) and 3D structures (**B**).

The molecular modelling was done with CS Chem3D Ultra, version 16.0, and execution took place by molecular orbital computations software (MOPAC) and molecular dynamics computations software (MM2 and MMFF94). The most likely sites for the interaction between CLA and TKI are found on the TKI, which have the highest electron density (Table 3). For verification of these sites’ participation, energy minimization was performed for one molecule of TKI with the number of CLA molecules obtained from the molar ratios. The CLA molecule was observed adjacent to the suggested sites with the highest electron density (Figure 6). Exceptionally in the case of OLA, the CLA molecule was adjacent to the carbonyl oxygen atom (O11), although it had lower electron density than those on the amide nitrogen atoms (N22 and N25). This information confirmed that CLA–TKI interactions happen through *n* → π* interactions. From the results of the molar ratio and computational molecular modelling, it was clear that these are the electron-donating sites on TKI molecules that are involved in generation of the produced CTCs with CLA.

### 2.6. Optimization of MW-SPA Conditions

**Figure 7:**
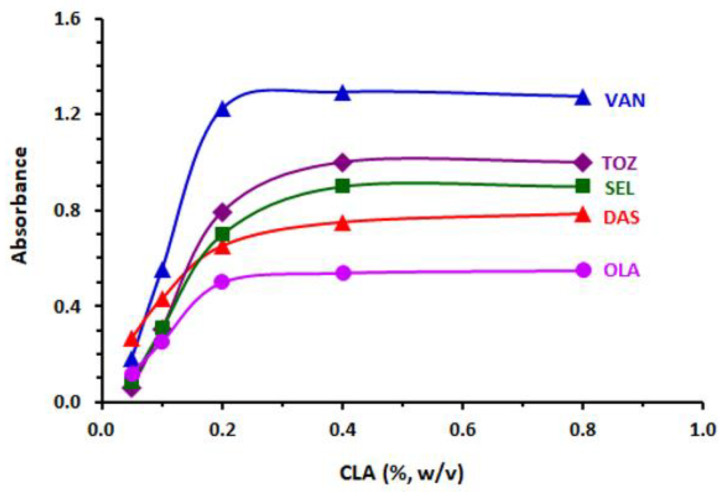
Effect of CLA concentration on its reaction with TKIs (200 µg/well). Measurements were carried out at 490 nm.

In order to have an excellent result in the 96-microwell assay plate, the experimental conditions were adjusted by adopting a “change one factor at a time” approach. Among the different tested solvents (Table 4), methanol was the optimum solvent, and it was used through the whole study, and measurements were recorded at 490 nm (the nearest filter to the λ_max_ of all investigated TKIs complexes of CLA). The observed results of changing CLA concentrations and the time of the reaction at room temperature (25 ± 2 °C) showed that the optimum CLA concentrations ranged from 0.2 to 0.8% (*w/v*), as shown in Figure 7. Similar experiments were conducted in order to optimize the reaction time, and it was discovered that the reaction was instantaneous; nevertheless, for obtaining the best reading precision, the measurements were carried out after 5 min from the starting point of the reaction. A summary of the condition ranges studied and the optimum value selected for the development of the proposed MW-SPA are included in Table 4.

### 2.7. Validation of MW-SPA

#### 2.7.1. Linear Range and Sensitivity

**Figure 8:**
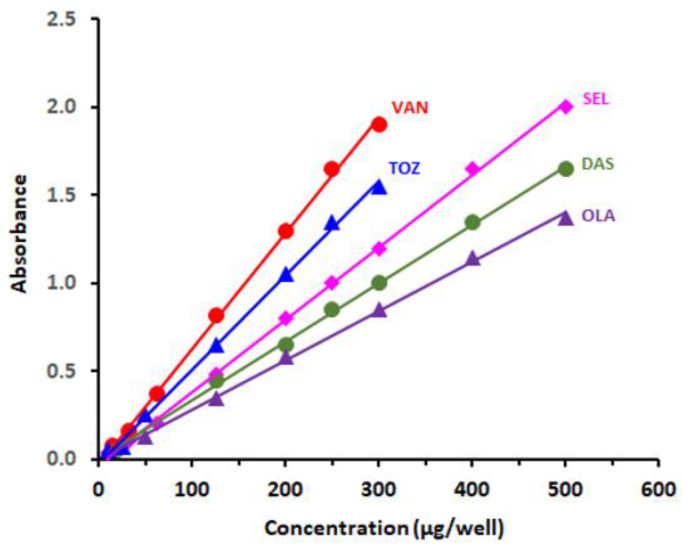
Calibration curves for determination of TKIs by the proposed 96-microwell-based spectrophotometric assay based on their reaction with CLA. Measurements were carried out at 490 nm.

The calibration curves were constructed (Figure 8) according to optimum conditions of the MW-SPA (Table 4), and the least square method was used for linear data regression. Calibration curves in the range of 10–500 µg/well (100 µL) were linear with excellent correlation coefficients. The limits of detection (LOD) and quantitation (LOQ) were determined based on the International Conference on Harmonization (ICH) guidelines [60]. The LOD and LOQ levels lay at 3.78–8.16 and 11.36–24.46 µg/well, respectively. A summary for the calibration and validation parameters of the current MW-SPA is given in Table 5.

#### 2.7.2. Precision and Accuracy

The accuracy of the proposed MW-SPA was determined utilizing samples of TKI solutions at various concentration levels (Table 6). The values of relative standard deviation (RSD) were 1.24–2.24 and 1.51–2.87% for intra- and inter-assay accuracy, respectively. The high precision of the method was proved by these low RSD values. The accuracy of the proposed method was assessed by the recovery studies. The recovery values ranged from 97.2 to 102.4% (Table 6), reflecting the accuracy of the assay.

#### 2.7.3. Robustness and Ruggedness

The robustness of the method (effect of small changes in the variables on its performance) was assessed [60]. The results of the test were found to be not significantly affected by small variations in the studied variables; the recovery values ranged between 97.5 and 102.3 ± 1.76 and 2.49%, respectively. This confirmed that the proposed test was convenient for routine TKI analysis.

Additionally, the ruggedness was tested by performing the method by at least two different analysts on three different days [60]. The results were reproducible since RSD values never exceeded 2.8%.

#### 2.7.4. Specificity and Interference

The advantage of the suggested MW-SPA is that measurements in the visible region are performed away from UV-absorbing interfering substances, which may be co-extracted from TKI-containing pharmaceutical formulations. Possible interference of additives in dosage forms was also studied. Mixing known quantity of TKI with different quantities of the familiar excipients was done to produce samples. These excipients included microcrystalline cellulose, magnesium stearate, sodium starch glyconate, colloidal silicon dioxide and anhydrous dibasic calcium phosphate. The results given in Table 7 showed that no interference was noted from any of the mentioned excipients with the suggested methods, as the recovery values ranged from 97.5 to 102.9%. The absence of interference with these excipients was caused by the organic solvent extraction of the TKI target from samples, where the excipients were not dissolved.

### 2.8. Application of MW-SPA in the Analysis of TKIs in Pharmaceutical Formulations

The successfulness of the validation results demonstrated that the suggested procedure was suitable for routine QC analysis of the investigated TKIs. The MW-SPA was used to determine TKIs in various pharmaceutical formulations, and the results are depicted in Table 8. The acquired mean values of the marked amounts were in the range of 98.6% to 103.1%. The results proved that the proposed MW-SPA was appropriate for assaying the investigated TKIs in their tablets.

In the present study, the detailed spectrophotometric investigations and quantitative analysis were given for five TKIs; however, other TKIs (more than 10) were tested in our laboratory for their ability to form CT complexes with CLA, and their results were positive. Their universal ability to form CT complexes was attributed to the fact that all TKIs contain electron-donating atoms in their chemical structure; that is the main requirement for formation of charge transfer complexes. In addition, we confirmed that all TKIs, regardless their chemical structures, did not absorb above 400 nm (the UV cut off wavelength) because they all were not colored. Furthermore, we confirmed that, even if the TKI molecule absorbed at above 400 nm, it could be analyzed by its reaction with CLA as long as its absorption spectrum did not extend to the maximum absorption peak of the complex (475–517 nm).

## 3. Experimental

### 3.1. Apparatus

A double beam ultraviolet–visible spectrophotometer with matched 1-cm quartz cells (UV-1800, Shimadzu Co. Ltd., Kyoto, Japan) was operated for the scanning of all the generated UV–visible spectra. An absorbance microplate reader (ELx808: Bio-Tek Instruments Inc., Winooski, VT, USA) powered by KC Junior software provided with the instrument was used.

### 3.2. Chemicals and Materials

All the investigated TKIs were procured from LC Laboratories (Woburn, MA, USA) and Weihua Pharma Co. Limited (Hangzhou, Zhejiang, China) and utilized as provided. Their purity was >99% (as claimed by the providing companies), and their solutions remained stable for at least 7 days under refrigeration. CLA was bought from BDH Chemicals Co. (Langenfeld, Germany). Transparent 96-microwell plates were procured from Corning/Costar Inc (Cambridge, USA). AdjusTable 8-channel pipettes were obtained from Sigma-Aldrich Chemicals Co (St. Louis, Missouri, USA). BRAND^®^ PP reagent tanks with lids for the pipettes were acquired from Merck KGaA (Darmstadt, Germany). The other reagents and solvents were of analytical grade (Fisher Scientific, California, CA, USA). The pharmaceutical formulations were caprelsa tablets (AstraZeneca, Cambridge, United Kingdom) labelled to contain 300 mg of VAN; sprycel tablets (Bristol Myers Squibb, New York, NY, USA) labelled to contain 50 mg DAS per tablet; and lynparza tablets (AstraZeneca, Cambridge, United Kingdom) labelled to contain 150 mg OLA per tablet. Laboratory-made tablets were prepared in the lab by combining individually accurate amounts (100 mg) of TOZ and SEL with 25 mg of each of starch, lactose monohydrate, microcrystalline cellulose and hydroxypropyl cellulose.

### 3.3. Preparation of TKIs Standard Solutions

Stock solutions of 5 mg/mL of SEL, VAN and OLA were obtained by dissolving 50 mg of the standard material in 10 mL of methanol, while 0.5 mg/mL of DAS and TOZ solutions were made by dissolving 5 mg of the standard material in 10 mL of methanol. These above-mentioned stock solutions were found to be stable over a period of 14 days when stored in the refrigerator.

### 3.4. Determination of Association Constants

A set of TKI solutions ranging from 1.79 × 10^−4^ to 1.85 × 10^−3^ M were swirled with a constant CLA concentration (4.8 × 10^−3^ M). The reaction was instant at room temperature (25 ± 2 °C). The absorbances of the colored solutions were recorded at their absorption peaks against exactly prepared reagent blanks. The measured absorbances were utilized to produce the plot of Benesi–Hildebrand [58] by plotting the values [A_0_]/A^AD^ versus l/[D_0_]. Linear regression analysis was shown for the data using the following Benesi–Hildebrand equation [60]:

$$\frac{\left[A_{0}\right]}{A^{\mathrm{AD}}}=\frac{1}{\varepsilon^{\mathrm{AD}}}+\frac{1}{K_{c}^{\mathrm{AD}}\cdot \varepsilon^{\mathrm{AD}}}\times \frac{1}{\left[D_{0}\right]}$$

where [A_0_] represents the CLA molar concentration (the acceptor); [D_0_] represents TKI molar concentration (the donor); A^AD^ represents the absorbance of the CTC reaction mixture formed; ε^AD^ represents the molar absorptivity of the CTC; and *Kc*^AD^ represents the formation constant of the complex (L mol^−1^). The intercept of the linear fitting equation was equivalent to 1/ε^AD^, and the formation constant was calculated from the derived value of ε^AD^ in addition to the slope of the equation.

### 3.5. Determination of CLA:TKI Molar Ratio

The spectrophotometric titration methods were applied in the current work [61]. Principal solutions of the investigated TKIs (2.5 × 10^−3^ M) and CLA (2 × 10^−2^ M) were readily prepared (i.e., molar concentration of CLA was 8 times bigger than that of TKI). Exceptionally, PEL concentration was (1 × 10^−3^ M) and that of CLA was 8 × 10^−3^ M. A set of master solutions of both the investigated TKIs and CLA were made to give molar ratios of TKI:CLA of 0.25:8. These solutions were always composed of constant TKI concentrations. The temperature was 25 ± 2 °C, and the corrected absorbances of the products were measured at 490 nm (after subtracting the readings of blanks that were treated similarly but using methanol instead of the sample) A graph was drawn by plotting the corrected measured absorbances versus the TKI:CLA molar ratio. From this graph, the molar ratio of the reaction was computed. The mole ratio corresponds to the point of intersection of the tangents of straight-line portions of the plots.

### 3.6. Preparation of TKI Tablets Solutions

The total amount of commercialized or synthetic tablets accounting for 50 mg of TKI (5 mg was used in case of DAS and TOZ) was placed in a 10-mL measuring flask, dissolved in a volume of 5 mL methanol, mixed thoroughly and sonicated for 5 min, completed to the mark with methanol, well shaken for 10 min and then filtered. The first portion of the filtrate was thrown away, and the exact volume of the filtrate was diluted with methanol. The final concentrations of TKIs ranged from 50 to 5000 μg mL^−1^.

### 3.7. Procedure of MW-SPA

Aliquots (100 microliters) of the standard or tablet sample solutions were composed of varying concentrations of TKI, ranging from 5 to 500 µg, and were placed to 96-well plates in addition to 100 microliters of 0.4% *w/v* of CLA solution. The reaction was carried out at 25 ± 2 °C for 5 min. Absorbances of the product were measured by the plate reader at a selected wavelength (490 nm). The blank wells were treated exactly the same as the other wells, except for the addition of 100 µL of methanol to each of them instead of the TKI solutions. Then, the absorbances of the samples were corrected by subtracting those of the blanks.

## 4. Conclusions

The present study found the CLA reagent to be a universal chromogenic reagent for TKIs. The experiment proved how the reaction proceeded, through formation of colored CTCs between CLA and TKIs. The reaction was used to develop novel MW-SPA for the five investigated TKIs. The proposed assay outperformed all the established assays for TKIs, since it could be applied for assaying all TKIs irrespective of the differences in their chemical structures. In the presented work, five TKIs were tested; however, the universal applicability of the proposed assay was supported by another study that was carried out in our laboratory [62]. Extended advantages of the suggested MW-SPA are the easiness of the procedure (simplicity), the use of affordable analytical reagents (economic), the requirement of minimal volumes of reagent and solvent (eco sustainable “green” approach) and high throughput. All these advantages make the suggested MW-SPA an effective universal TKI assay for routine QC laboratory use.

## Figures and Tables

**Table 1:** The investigated TKIs with names, abbreviations, IUPAC names, molecular formulae and molecular weights.

TKI Name	Abbreviation	IUPAC Name	Molecular formula	Molecular Weight
Seliciclib	SEL	(2*R*)-2-{[6-(benzylamino)-9-(propan-2-yl)-9*H*-purin-2-yl]amino}butan-1-ol	C_19_H_26_N_6_O	354.46
Vandetanib	VAN	*N*-(4-bromo-2-fluorophenyl)-6-methoxy-7-[(1-methylpiperidin-4-yl) methoxy]quinazolin-4-amine	C_22_H_24_BrFN_4_O_2_	475.40
Tozasertib	TOZ	*N*-[4-[4-(4-methylpiperazin-1-yl)-6-[(5-methyl-1*H*-pyrazol-3-yl) amino] pyrimidin-2-yl] sulfanylphenyl]cyclopropanecarboxamide	C_23_H_28_N_8_OS	464.59
Dasatinib	DAS	*N*-(2-chloro-6-methylphenyl)-2-[[6-[4-(2-hydroxyethyl) piperazin-1-yl]-2-methylpyrimidin-4-yl]amino]-1,3-thiazole-5-carboxamide	C_22_H_26_ClN_7_O_2_S	488.01
Olaparib	OLA	4-[[3-[4-(cyclopropanecarbonyl)piperazine-1-carbonyl]-4-fluorophenyl] methyl]-2*H*-phthalazin-1-one	C_24_H_23_FN_4_O_3_	434.47

**Table 2:** Spectrophotometric parameters of the CT complex reaction of CLA with TKIs.

TKIs	λ_max_ (nm)	ε_max_ × 10^3^ (L mol^−1^ cm^−^^1^)	Band Gap Energy Value (eV)	Association Constant (L mol^−1^ × 10^8^)	DG^0^ (J mol^−1^ × 10^4^)	Molar Ratio [TKI:CLA]
SEL	497	0.83	1.90	2.00	−4.74	1:2
VAN	517	1.54	1.90	2.18	−4.76	1:2
TOZ	512	1.47	1.90	1.13	−4.60	1:2
DAS	475	1.09	1.90	2.30	−4.77	1:2
OLA	494	0.73	2.14	1.97	−4.73	1:1

**Table 3:** The molar ratios of the reaction of TKIs with CLA, types of atoms proposed as site(s) of interaction on TKIs molecules and charges on these atoms.

TKI	TKI:CLA Molar Ratio	Atom Type(s) Proposed as Site(s) of Interaction ^a^	Charge ^b^
SEL	1:2	(N10): Enamine or aniline nitrogen, delocalized lone pair of electrons	−0.8691
		(N21): Enamine or aniline nitrogen, delocalized lone pair of electrons	−0.8691
VAN	1:2	(N11): Enamine or aniline nitrogen, delocalized lone pair of electrons	−0.6
		(N23): Amine nitrogen	−0.81
TOZ	1:2	(N2): Aromatic 5-ring nitrogen	−0.7068
		(N14): Enamine or aniline nitrogen, delocalized lone pair of electrons	−0.8382
		(N17): Amine nitrogen	−0.81
DAS	1:2	(N28): Enamine or aniline nitrogen, delocalized lone pair of electrons	−0.8382
		(N29): Amine nitrogen, *N*-hydroxyethyl	−0.81
		(O30): Alcohol or ether oxygen	−0.68
OLA	1:1	(O11): Carbonyl oxygen in amide	−0.57
		(O21): Carbonyl oxygen in amide	−0.57
		(N22): Amide nitrogen	−0.6602
		(N25): Amide nitrogen	−0.6602

^a^ These sites of interactions are denoted on the chemical structures of the TKIs (Figure 1). ^b^ The negative sign indicates negative electron density.

**Table 4:** Optimization of experimental conditions for the 96-microwell spectrophotometric assay for TKIs based on their CT reaction with CLA.

Condition	Studied Range	Optimum Value ^a^
CLA conc. (%, *w/v*)	0.01–0.8	0.4
Solvent	Different ^b^	Methanol
Reaction time (min)	0–40	5
Temperature (°C)	25–60	25
Measuring wavelength (nm)	400–800	490

^a^ Optimum values were used for all TKIs. ^b^ Solvents used were acetonitrile, methanol, ethanol, acetone, propanol, butanol, dichloroethane, dichloromethane, chloroform, diethyl ether, benzene and dioxan.

**Table 5:** Calibration parameters for the analysis of TKIs by the 96-microwell spectrophotometric assay based on their CT reaction with CLA.

TKIs	Linear Range ^a^	Intercept	SDa ^b^	Slope	SDb ^b^	r ^b^	LOD ^a^	LOQ ^a^
SEL	10–500	0.0034	0.52 × 10^−2^	0.0041	1.5 × 10^−3^	0.9995	4.12	12.38
VAN	15–300	0.0028	0.78 × 10^−2^	0.0067	1.2 × 10^−3^	0.9997	3.84	11.52
TOZ	10–300	0.0081	0.60 × 10^−2^	0.0053	0.6 × 10^−2^	0.9998	3.78	11.36
DAS	20–500	0.0056	0.68 × 10^−2^	0.0033	0.1 × 10^−2^	0.9993	6.78	20.32
OLA	25–500	0.0065	0.64 × 10^−3^	0.0028	4.3 × 10^−3^	0.9996	8.16	24.46

^a^ Values are in µg/well. ^b^ SDa = standard deviation of the intercept, SDb = standard deviation of the slope, r = correlation coefficient.

**Table 6:** Precision and accuracy of the proposed 96-microwell spectrophotometric assay for TKIs via their CT reactions with CLA.

TKIs	Relative Standard Deviation (%) ^a^		Recovery (% ± SD) ^a^
	Intra−Assay, *n* = 3	Inter−Assay, *n* = 3	
SEL	2.12	2.25	101.7 ± 2.3
VAN	1.24	1.51	102.3 ± 2.2
TOZ	1.53	2.11	97.6 ± 1.9
DAS	2.24	2.87	102.4 ± 2.3
OLA	2.01	2.54	97.2 ± 2.6

^a^ Values are the means of three determinations.

**Table 7:** Analysis of TKIs in the presence of the excipients in solid pharmaceutical tablets by the proposed 96-microwell spectrophotometric assay based on their CT reactions with CLA.

Excipient ^b^	Recovery (% ± SD) ^a^				
	SEL	VAN	TOZ	DAS	OLA
MCC (50) ^c^	100.9 ± 0.8	101.3 ± 0.56	99.7 ± 1.1	99.5 ± 1.2	101.5 ± 1.2
CSD (10)	97.5 ± 1.5	98.6 ± 1.3	100.6 ± 0.9	100.9 ± 0.9	100.1 ± 1.8
ADCP (5)	101.3 ± 0.9	101.3 ± 1.2	98.8 ± 1.4	102.3 ± 1.7	98.8 ± 1.9
SSG (5)	101.2 ± 0.8	102.9 ± 0.8	99.2 ± 1.6	101.6 ± 1.3	99.2 ± 1.6
MS (5)	99.4 ± 1.2	101.8 ± 1.5	97.9 ± 1.9	100.4 ± 1.4	102.1 ± 1.4

^a^ Values are means of three determinations. ^b^ Abbreviations: MCC = microcrystalline cellulose, CSD = colloidal silicone dioxide, ADCP = anhydrous dibasic calcium phosphate, MS = magnesium stearate. ^c^ Figures in parenthesis are the amounts in mg added per 50 mg of TKI.

**Table 8:** Determination of TKIs in their pharmaceutical formulations by the proposed MW-SPA based on their CT reaction with CLA.

Taken Conc. (μg/well)	Recovery (% ± SD) ^a^				
	Caprelsa Tablets (300 mg VAN)	Sprycel Tablets (70 mg DAS)	Lynparza Tablets (150 mg OLA)	LM TOZ Tablets ^b^ (100 mg TOZ)	LM SEL Tablets ^b^ (100 mg SEL)
50	102.4 ± 1.3	100.6 ± 1.3	97.9 ± 2.2	101.4 ± 1.8	99.1 ± 1.7
100	98.9 ± 1.2	101.9 ± 1.6	99.2 ± 1.4	103.1 ± 2.6	101.5 ± 1.4
150	101.8 ± 1.8	100.8 ± 1.1	101.3 ± 1.6	99.3 ± 1.2	98.6 ± 1.9
250	99.7 ± 2.1	99.5 ± 1.8	100.6 ± 1.5	101.3 ± 2.8	100.8 ± 1.6

^a^ Values are mean of three determinations. ^b^ LM means laboratory made.

## Data Availability

Data is contained within the article.

## Sample Availability

Samples of the compounds are not available from the authors.
